# Supplementary material for: Self-controlled practice and nudging during structural learning of a novel control interface
Source: PLoS One. 2020 Apr 14;15(4):e0223810. doi: 10.1371/journal.pone.0223810 (PMC7156047; doi:10.1371/journal.pone.0223810)
Supplement: S1 Data — (ZIP) [file pone.0223810.s001.zip › Description of DataFiles.docx]

Description of DataFiles

Attached are 4 JASP files associated with the analyses in the manuscript. These are split by dependent variable

1. Movement time analyses – BoMI_MT.jasp
2. Path length analyses – BoMI_PL.jasp
3. Selection of ‘difficult’ targets – BoMI_DiffTarget.jasp
4. Repetition of targets – BoMI_RepTarget.jasp

Column names represent the corresponding blocks. E.g. MT_5 – Movement time in training block 5, MT_Pre = Movement time in Pre-test etc.
