# Supplementary material for: Self-controlled practice and nudging during structural learning of a novel control interface
Source: PLoS One. 2020 Apr 14;15(4):e0223810. doi: 10.1371/journal.pone.0223810 (PMC7156047; doi:10.1371/journal.pone.0223810)
Supplement: Attachment — Submitted filename: Response letter_v1.docx [file pone.0223810.s002.docx]

We thank the academic editor and reviewer for their insightful comments. Our responses to each of the comments are provided below. We think these changes have significantly improved the clarity of the manuscript and addressed all concerns.

Thank you.

Please refer to the following color scheme:

Reviewers’ original comments are in BLACK

Authors’ responses are in BLUE

Corresponding changes in the manuscript are italicized and highlighted in *GREY*

----------------------------------------------------------------------------------------------------------------

Additional Editor Comments (if provided):

It was not clear to me how the difficulty of the targets was equated in the nudge group. As I understand, the targets were made bigger or smaller by 25%, that choice was based on participant’s performance at pre-test or mid-test. This number (25%) seems rather arbitrary: was the error in average 25% larger for the most difficult target? The discussion does mention this issue but a stronger case could be built by collecting data for another study using the method described in the discussion.

We thank the editor for this important comment. The choice of 25% was not based on the magnitude of the error, instead it was mainly selected so that it serve as a ‘nudge’ (i.e. a slightly larger target would increase the chance that it would be selected more often; however if the target were made very large it would no longer be a nudge as it essentially eliminates all other choices). Indeed, this manipulation was successful as we found that the Nudge influenced target selection. Therefore, in spite of this 25% being a pre-defined choice, it had the desired effect on the experiment.

The comment about running an experiment using the method described in the discussion is in our future plans – we are evaluating different methods of nudging to develop this as a further question. However, given that this is a separate research question in itself, we think that this distracts from the main focus of the current manuscript. So, we plan to do this work in the future but are not including it in the current manuscript.

We have now included the justification for the 25% increase in the manuscript as follows:

*The 25% increase in radius was chosen to be a ‘nudge’ – i.e. it was large enough that participants could perceive it as the biggest (or smallest) target, but not so large that it essentially forced participants to choose (or eliminate) that target.*

Some of the conclusions rely on the lack of statistical significance. I would recommend using either a parametric test of equivalence (e.g., Toster by Lakens) or providing Bayes Factors. Since you are using JASP, I would think it should be very easy to determine the strength of the null hypothesis.

We thank the editor for this point. We have now added Bayes factors to the central comparison for the main dependent variable (i.e. the main effect of Group and the Group x Block interaction in the Movement Time analyses). As seen from the results, the Bayes Factors (>10 in all cases) highly favor the null hypotheses (i.e. that the groups are not different), supporting the conclusions we had made.

Statistics section:

*Because the focus was on the comparison between groups, we also report Bayes Factors for the Group and Group x Block interactions in the ANOVA. We used the default prior settings in JASP and used the ‘analysis of effects’ table under the BFexclusion column, which indicates the change from prior to posterior exclusion odds [35].*

Results: - Movement Time

*To examine the strength of these null results, we also computed Bayes factors. The BFexclusion for the group effect was 13.534 and group x block interaction was 59.557 indicating that the data strongly support excluding these terms (i.e. they had no effect).*

Results – Path length

*To examine the strength of these null results, we also computed Bayes factors. The BFexcl for the group effect was 10.890 and group x block interaction was 43.044 indicating that the data strongly support excluding these terms (i.e. they had no effect).*

Journal Requirements:

When submitting your revision, we need you to address these additional requirements.

1. Please ensure that your manuscript meets PLOS ONE's style requirements, including those for file naming. The PLOS ONE style templates can be found at

[http://www.journals.plos.org/plosone/s/file?id=wjVg/PLOSOne_formatting_sample_main_body.pdf](https://urldefense.com/v3/__http:/www.journals.plos.org/plosone/s/file?id=wjVg*PLOSOne_formatting_sample_main_body.pdf__;Lw!!Ls64Rlj6!mBEMLMC5zFGZR06UdNWZgEoXDAAfRlwfCWVY2CruV30dMaXRL7UKtBjMgfLzUuxdUPdw-V-9xOU$) and [http://www.journals.plos.org/plosone/s/file?id=ba62/PLOSOne_formatting_sample_title_authors_affiliations.pdf](https://urldefense.com/v3/__http:/www.journals.plos.org/plosone/s/file?id=ba62*PLOSOne_formatting_sample_title_authors_affiliations.pdf__;Lw!!Ls64Rlj6!mBEMLMC5zFGZR06UdNWZgEoXDAAfRlwfCWVY2CruV30dMaXRL7UKtBjMgfLzUuxdUPdwZXBthjY$)

We have now made all necessary formatting changes to conform to PLOS ONE’s style requirements as outlined in the links above.

2. Please ensure that you refer to Figure 4 in your text as, if accepted, production will need this reference to link the reader to the figure.

We have now referred to Figure 4 in the relevant sections of the Results.

[Note: HTML markup is below. Please do not edit.]

Reviewers' comments:

Reviewer's Responses to Questions

**Comments to the Author**

Reviewer #1: # General comments

The experiment aimed to investigate whether a practice condition controlled by the learner promotes benefits for a specific case of learning (structural learning). An experimenter imposed random practice schedule served as a control group for two self-controlled groups, one without and another with “nudge”. “Nudging” in the sensorimotor task used by the authors consisted of displaying larger targets in positions identified to be more difficult for the learners to “reach” (by controlling a virtual cursor).

The introduction presents the problem clearly and the Method contains all information necessary to understand the experimental task and procedures. The statistical analysis is adequate and presented in detail, as is the results section.

We thank the reviewer for the positive comments.

Nevertheless, I have some concerns related to the discussion of the findings.

The crucial aspect for me is that the “random practice” condition does not allow to control for the effects of the learners autonomy (which is a limitation addressed by the authors in the discussion) and, most critical, this practice condition does not control for the effects of practice organization. Specifically, the practice organization “produced” by each learner as a result of their decision making during practice does not compare to the pseudo random practice performed by the random group. This aspect is critical because it changes important interpretations presented in the discussion.

We agree with the reviewer that we did not control for autonomy using a yoked group but this was not the central focus of the manuscript (we have noted this as a limitation and refrained from making statements about the effect of autonomy). However, the question of practice organization is the central focus here (i.e. is a self-selected sequence better for learning compared to a random sequence?). Therefore we are not sure what the reviewer means by “the practice condition does not *control* for effects of practice organization” – we did not control for this precisely because our goal was to examine if influencing the practice organization (random/self-selected/nudge) results in differences in learning.

\# Specific points:

“A feature of self-controlled learning that may be problematic here is that although it may benefit autonomy, it typically reduces the random structure of practice because participants tend to engage in more ‘blocked’ practice by repeating targets (24).”

“In the context of structural learning, self-controlled practice may create a potential tradeoff - participants may tend to focus excessively on improving performance on the training targets (as indicated by the increased repetition and avoidance of the difficult targets in the Strategy analyses), however, this focus on short-term performance may result in more ‘blocked’ practice, which could negate some of the other benefits of self-controlled practice.”

I recommend that the authors search the literature concerning this point, since there is evidence showing that this behavior actually depends on the instruction provided (i.e. participants only “tend to engage in more ‘blocked’ practice” in the absence of instructions beyond the ones describing the task goal).

We thank the reviewer for raising this point. We did a further literature search and found that in addition to one article we had cited, two more articles (Safir et al., 2013; Hodges et al., 2011) using the self-selected practice sequence found exactly the same finding (i.e. that participants engage initially in blocked practice and later transition to a more ‘random’ like practice schedule). We have now added these references in the text with the caveat that the typical interpretation of this blocked practice is ‘being beneficial’ to learning because it matches the challenge-point.

We were unable to find an article that links this to the presence or absence of instructions. If the reviewer feels this is critical, we would appreciate it if the reviewer could point us to this study.

*A feature of self-controlled learning that may be problematic here is that although it may benefit autonomy, it typically reduces the random structure of practice because participants typically tend to engage in more ‘blocked’ practice by repeating targets, at least early on in learning [23–26]. Although one view is that this initial blocked practice is beneficial because it matches their challenge point [27] , a downside is that it may foster more ‘instance-based’ learning (i.e. how to solve a particular variation), and may potentially be detrimental to ultimately learning the underlying rule or schema.*

----

“The goal of the study was to address the role of self-controlled practice in a structural learning task.”

I suggest rephrasing “structural learning task”, because the ambiguity may suggest that “structural learning” refers to an attribute of the task.

We thank the reviewer for bringing our attention to this point. We have now re-phrased the sentence as follows:

*The goal of the study was to address the role of self-controlled practice and nudging during structural learning of a novel task.*

----

“One trivial possibility for these non-significant results is simply that any potential differences between groups was eliminated by a ‘floor effect’ in terms of the performance – i.e. movement times had reduced to a minimum possible limit by the end of training. However, we consider this unlikely as an explanation since the mid-tests (which were done in the middle of the training session) also showed the same patterns as the post-test.”

This argument is not clear to me. I would recommend rewriting it.

We apologize for a lack of clarity in this statement. Basically, we wanted to state that the argument for a ‘floor effect’ (i.e. that movement times had saturated to a lower limit) does not seem likely as the results hold even during the mid-test (when the movement times have not reached a floor yet). We have revised it as follows:

*A possible explanation for these non-significant results in the post-test is that each group experienced a “floor effect” i.e. that reduction in movement times had reached a limit by the end of training. However, we consider this explanation unlikely since the same pattern results are seen even in the mid-test when the movement times were still decreasing.*

----

“Approaches such as ‘restricted’ self-control, where participants face a mix of self-controlled and experimenter-imposed conditions may provide the optimal learning environment in such cases (8)”

The task used in the cited study (8) was not similar to the one employed in the study under review. Considering the amount of studies investigating self-controlled sensorimotor learning not included in their paper - which, I thought, were not included because of the specificities of their task* -, the criteria used by the authors to select which research to cite is sometimes unclear.

* “...these have been primarily used in rather well-learned tasks (such as key pressing or throwing) where the underlying schema may already be present through prior experience. In contrast, our focus in this study was to use a novel virtual task where the structure could only be learned through practice.”

We thank the reviewer for the comment. We want to clarify that our main criteria for selecting papers was not the task itself, but that they manipulate the practice schedule (and not KR or other aspects which is the predominant manipulation in the literature). However, we have also cited a few original and review articles for the overall effects of self-controlled learning in the introductory paragraph.

The cited paper (Andrieux et al., 2016) best highlights one of the weakness of self-controlled practice even though it was not in the context of a structural learning task. The authors in this paper state “More specifically, learners engaged in a complete self-controlled (SC) condition tended to adopt a more comfortable, lower nominal level of task difficulty throughout the acquisition phase in order to increase the number of intercepted targets (p. 61).” As far as we can tell, this ‘downside’ to self-controlled learning is not mentioned in earlier articles.

----

“One reason for this null result might be that we only evaluated target difficulty twice during the entire practice schedule - at the onset of practice and at the halfway mark (i.e. at the pre-test and mid-test). A more frequent update of task difficulty (e.g., once per training block) may have been more effective to ensure that participants were practicing on the most difficult target for them at that time.”

This would mean that any conclusions concerning "nudging" would be inappropriate, since the method did not allowed the adequate manipulation of the independent variable. If I'm mistaken, maybe the argument needs some adjustments.

We thank the reviewer for the comment. We want to emphasize the Nudge in our experiment did have the desired effect in terms of influencing the target selection. However, because it did not have any effect on learning, we suggest future study designs can use “real-time” nudging (by more frequently determining which is the more difficult target at each time point), and by adjusting the size in a more proportional way (say according to Fitts Law).

We have now rephrased this point to make sure that what we are suggesting are ‘improvements’ to the Nudge (and not that it failed in the current experiment)

*Results showed that the nudge worked as a manipulation – i.e. the Nudge group did successfully alter the strategy relative to the Self-selected group in terms of increasing the choice of the difficult target initially in learning. However, our results showed no reliable effect of this manipulation on any of the performance metrics relative to the Self-selected group which was not nudged.*
