# Supplementary material for: Self-controlled practice and nudging during structural learning of a novel control interface
Source: PLoS One. 2020 Apr 14;15(4):e0223810. doi: 10.1371/journal.pone.0223810 (PMC7156047; doi:10.1371/journal.pone.0223810)
Supplement: Attachment — Submitted filename: Response letter_v2.docx [file pone.0223810.s003.docx]

We thank the Editor for taking the effort to review our previous submission in the absence of the reviewer and suggesting the reference we missed. We have now incorporated this in the manuscript.

Thank you.

Mei-Hua Lee

Thank you for submitting your manuscript to PLOS ONE. After careful consideration, I believe the paper can be accepted but there is one point that I would appreciate if the authors could check before I make a final decision. Therefore, I invite you to submit a revised version of the manuscript that addressing the point raised below.

==============================
Unfortunately, due to an illness, the reviewer who provided comments on the original submission could not assess your rebuttal letter. Rather than seeking another reviewer, I took on the responsibility of assessing your answers myself. I think for the most part they are fine and I would have no further concerns. With regards to one point made by the Reviewer about instructions, I wondered whether she was referring to knowledge about the testing conditions. A few years back, I published a paper with colleagues showing that knowledge of how testing would occur after practice affects how they organized practice, improving learning (DOI: [10.1016/j.humov.2012.11.008](https://urldefense.com/v3/__https:/doi.org/10.1016/j.humov.2012.11.008__;!!HXCxUKc!n9pfV1yVNJxFuvfZX059yJc3WElrCZnnEQ3wxaVZOqyS0TCIZIqqT-_SERjy$)). I honestly don’t know if the reviewer had this type of work on mind or something else, but if you find it relevant to your discussion then you may want to consider that work. Of course, citing it or not has no impact on the paper’s acceptance and the authors are free to ignore this suggestion if they believe it doesn’t tackle the reviewer’s concern or doesn’t improve the paper's narrative. I will be ready to make a quick decision upon receiving your revised manuscript.

==============================

We thank the Editor for this comment. In our experiment, we had given participants the “learning goal” (i.e. that they would eventually have to learn to move to all 8 targets) – so we think this makes the findings even stronger. We have now added this reference in the manuscript.

At the end of the Methods:

Participants in the Self-selected and the Nudge groups were given the learning goal [34], i.e. they were instructed that while they had choice on what target to select on trials during training, they would be evaluated on how well they could do all targets at the end of training.

In the Discussion:

These results suggest that even when participants are given the overall learning goal (in this case, participants were told that they would be evaluated on all targets at the end of training) [34], self-controlled practice schedules may not always be optimal in terms of practice structure, especially in the context of learning novel tasks.
